# Eufemiusz J. Herman (1892–1985)

**DOI:** 10.1007/s00415-019-09428-4

**Published:** 2019-06-15

**Authors:** Anita Magowska

**Affiliations:** Poznan University of Medical Sciences, Poznan, Poland

**Figure 1:**
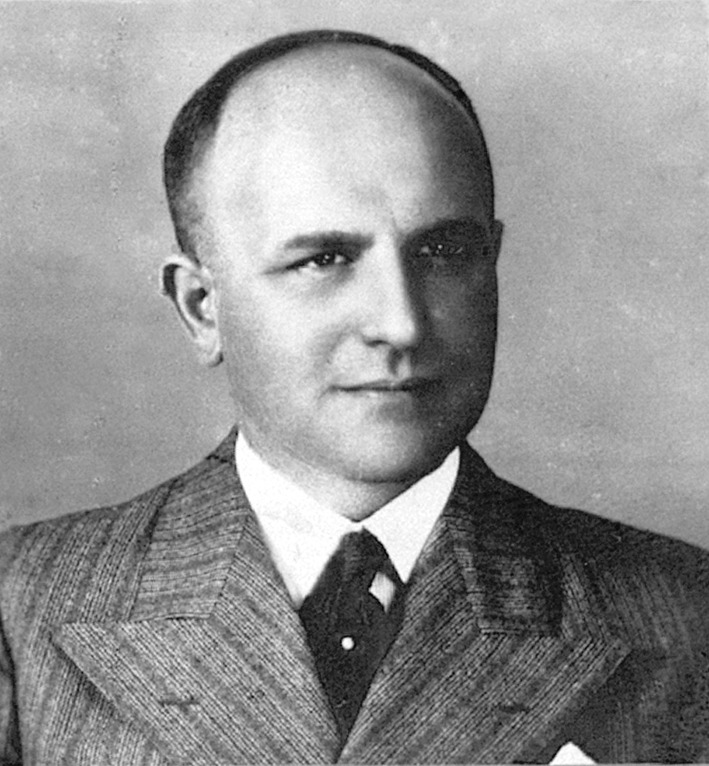
Eufemiusz J. Herman (by courtesy of the Main Medical Library in Warsaw)

Eufemiusz Józef Herman made many contributions to neurology, including the descriptions of previously undetected symptoms and syndromes, for example, the rare post-traumatic craniocerebral syndrome of vasomotor origin with livedo racemosa, pyramidal and extrapyramidal signs, speech disorders, hypertension, and progressive dementia [1] which now bears his name (Herman syndrome) [2] (Fig. 1).

Born on 29 September 1892 in the town of Tomaszow Mazowiecki in Russian Poland (from 1795 to 1918 Poland was partitioned by Austro-Hungary, Prussia, and Russia), E. J. Herman owed his interest in the natural sciences and medicine to his parents, Jakub and Helena née Krysztal, who were both teachers in Jewish primary schools. During his medical studies in Lvov and then in Krakow, he volunteered to work in a histology unit and then in neurological clinics. After obtaining the doctor title of the medical sciences (a title with centuries-old tradition) diploma in 1918, he joined the Polish Army and participated in the Polish–Soviet War.

Released from military service in 1922, he started work in the Neurology Department of the Jewish Hospital in Warsaw, headed by Edward Flatau. Under his supervision, Herman learned to conduct subtle clinical observations and associate perceived reflexes with the locus of a disease in the brain. He was quick to learn and soon took charge of the hospital-based neurological outpatient clinic. From 1922 until the outbreak of the Second World War, he also worked in the Laboratory of Neurobiology headed by Flatau and, after his death in 1932, by Kazimierz Orzechowski in the Nencki Institute in Warsaw. In 1932, he became successor to Flatau in the Jewish Hospital by way of open competition.

In April 1939, Herman joined the Polish Army again and helped to defend Poland during the German invasion in September. After defeating Poland, the Germans established a ghetto in Warsaw and relocated the Jewish Hospital there. Herman, who was still working in the hospital, treated many starving Jews suffering from typhus, noticing new neurological symptoms in them. When the Germans dissolved the ghetto, taking its residents away to the death camp in Treblinka, Herman managed to escape. Until the end of the war, he was helped by Polish farmers and lived in hiding.

After the war, Herman was appointed director of the ‘Kochanowka’ Psychiatric Hospital near Lodz, but in 1946 resigned to benefit from two prestigious grants. The Svenska Institute gave him a two-month internship in the Neurosurgical Clinic of Herbert Olivecrona in Stockholm and Columbia University awarded him a six-month internship at the Neurological Clinic in New York [3]. During his stay in the US, Herman shared the knowledge he had obtained in the ghetto, giving a lecture on the neurological forms of typhus for doctors from the Neurological Clinic of Cornell University [4].

In 1946, Herman also obtained a postdoctoral degree and as associate professor became head of the newly created Neurological Clinic of the University of Lodz. However, in 1956, new laws were introduced in Poland, which required professors to have a doctoral degree based on a dissertation. There was no way out; the Professor had to be admitted to a PhD program. Reviewers gave a high mark to his thesis on the tonic plantar reflex and its significance for the localization of lesions in the frontal lobes [5]. He retired 7 years later. He died on 8 May 1985, survived by his wife, Roza Maria née Lubinska, a leading Polish chess player of Jewish origin. Sadly, their only daughter, Krystyna, was killed by Germans in 1944 [6].

The essence of Herman’s talent as a neurologist was his clinical sense, the skill of a doctor that is now currently being pushed into the background by laboratory tests and imaging methods of diagnosis. He was first to describe, inter alia: the hypogastric-erection symptom in some brain tumours (1935); the follow-up symptom in hemiparesis (1946); neurological syndromes in typhus (1947); nuchal-toe sign in meningitis (1949, the so-called Herman sign); intermittent reflexes in myasthenia gravis (1949); chronic disseminated rheumatic encephalomyelitis (1954); the symptom of the narrowing of the palpebral fissure when looking aside, exacerbated by hidden paresis of the levator palpebrae superioris muscle of the upper eyelid (1959); the ischaemic test in myasthenia gravis (1962); and the symptom of upper eyelid retraction in internuclear ophthalmoplegia (1976).

This prolific author published a few monographs, among others on sciatica, headaches (1950), inflammatory diseases of the brain (1952), congenital, early acquired and familial nervous system diseases (1954), Polish neurologists (1958), and the history of neurology in Poland (1975) [3]. His handbook on the diagnosis of nervous system diseases (1961) was reprinted five times [7]; another one, on neurological syndromes in internal diseases, was published in Russian [8] and German [9]. He was a co-founder and president of the Polish Neurological Society and a long-time editor of the journal ‘Neurologia, Neurochirurgia i Psychiatria Polska’ [Polish Neurology, Neurosurgery and Psychiatry]. He received an honorary degree from the Medical University of Lodz.

Enthusiastic about the history of neurology, Herman was active within the Commission of the History of Neurology of the World Federation of Neurology, an organisation of which he was vice chairman [3, 10]. He was also a member of the Neurological Section of the Royal Society of Medicine and the Association of British Neurologists, and other international societies [3].
